# Th17 and Th1 Lymphocytes in Oligoarticular Juvenile Idiopathic Arthritis

**DOI:** 10.3389/fimmu.2019.00450

**Published:** 2019-03-14

**Authors:** Laura Maggi, Alessio Mazzoni, Rolando Cimaz, Francesco Liotta, Francesco Annunziato, Lorenzo Cosmi

**Affiliations:** ^1^Department of Experimental and Clinical Medicine and DENOTHE Center, University of Florence, Florence, Italy; ^2^Anna Meyer Children's Hospital and University of Florence, Florence, Italy

**Keywords:** Th17, Th1, CD161+ T cells, TNF-α, synoviocytes

## Abstract

In the last years much attention has focused on the Th17 and Th1 phenotypes and on their pathogenic role in juvenile idiopathic arthritis, investigating how the cytokines produced by T helper cells act on resident cells on the synovia and which signal transduction pathways regulate Th17 cells proliferation and plasticity. In this context, an important milestone was represented by the identification of the non-classic Th1 phenotype, developed from the shift of Th17 cells. The cytokine TNF-α, beyond its well-known proinflammatory activity is involved in this process and this is one of the reasons why the TNF-α inhibitors are widely used in the treatment of juvenile idiopathic arthritis patients.

## Introduction

Juvenile idiopathic arthritis (JIA) is one of the most common chronic conditions of childhood, comprising several forms of arthritis characterized by persistent joint inflammation for at least 6 weeks, with an onset before the age of 16 years and with unknown cause (1, 2). The term JIA covers seven pathologic conditions that differ for clinical presentation, disease course and treatment response; in particular it includes systemic arthritis (sJIA), oligoarthritis, polyarthritis (both rheumatoid factor positive or negative), psoriatic arthritis (JPsA), enthesitis-related arthritis (ERA), and undifferentiated arthritis (1, 2). Although the cause of disease is unknown, immune cells, including T and B lymphocytes, infiltrate the synovial membrane of inflamed joints, suggesting that the adaptive immune system is involved in the pathogenesis of JIA (3). Human effector CD4+ T lymphocytes can be classified in three main subsets based mainly on their immunological functions, their cytokines production profile and their typical transcription factor expression (4). Th1 lymphocytes express the transcription factor T-bet, produce interferon (IFN)-γ, and defend the body from intracellular infections. Th2 cells express the transcription factor GATA-3, produce type 2 cytokines (interleukin (IL)-4, IL-5, IL-9, and IL-13) and are important to protect against helminths (5, 6). Finally, the Th17 subset produce IL-17A, IL-17F and IL-22 (7–10), express the transcription factor ROR-γT (11–13) and the lectin receptor CD161 (14), as typical surface marker. Beyond their protective role against extracellular bacterial and fungal infections, Th17 cells have been demonstrated to be important in the pathogenesis of several autoimmune and inflammatory diseases, including multiple sclerosis, inflammatory bowel disease (IBD), psoriasis, rheumatoid arthritis (RA), and JIA (15, 16). In humans, Th17 lymphocytes are included within the CD161+ cell fraction of circulating and tissue-infiltrating CD4+ T cells, and they develop from a CD161+ T cell precursor found in umbilical cord blood and neonatal thymus (14, 17, 18).

Since JIA can be considered as an immune-mediated disorder, the pharmacologic therapy is essentially based on immunosuppressive drugs, at least when the usage of non-steroidal anti-inflammatory drugs (NSAIDs) does not control symptoms. Among these disease-modifying antirheumatic drugs (DMARDs) methotrexate (MTX), is considered the first line treatment, since its positive clinical effects are associated with low toxic effects (19). For those patients with a suboptimal response to non-biologic DMARDs, in particular those with polyarthritis, the usage of biologic drugs such as tumor necrosis factor-α (TNF-α) inhibitors (etanercept, infliximab and adalimumab), IL-1 inhibitors (anakinra, canakinumab, and rilonacept), IL-6 inhibitor (tocilizumab), CD20/B-cell targeted (rituximab) and T-cell co-stimulatory signal blocker (abatacept), has been proven to be effective (19–22).

## T Helper Effector Cells in Oligoarticular JIA

The synovial membrane of JIA inflamed joints shows high degree of infiltrating mononuclear cells, including T and B lymphocytes, dendritic cells and macrophages (3, 18, 23). Among T cells, Th1 are the most represented since these cells can migrate in the synovia in response to the chemokine CXCL10 (24). For this reason, these cells were thought to play a key role in the pathogenesis of oligoarticular JIA (25, 26), at least until 10 years ago. More recently, after the identification of the Th17 subset, many experimental data suggested their potential pathogenic role both in adult and childhood arthritis as well as in other inflammatory and autoimmune diseases (27, 28). Indeed, increased levels of IL-17A and of the transcription factor ROR-γT, as well as of Th17 cells were reported in the synovial fluid (SF) of oligoarticular JIA patients (26, 28–31).

Moreover, Th17 cells have been demonstrated to be pathogenic in several murine models of chronic inflammatory disorders (16), such as experimental autoimmune encephalomyelitis (7), collagen-induced arthritis (32), and IBD (10–12).

However, despite their supposed pathogenicity, Th17 are very rare at inflammatory sites if compared to Th1 cells (28, 33). A first explanation for this rarity is a self-regulatory mechanism that controls Th17 cells clonal expansion. In particular, ROR-γT favors the up-regulation of the interleukin (IL)-4 induced gene 1 (IL4I1), which encodes an l-phenylalanine oxidase that down-regulates CD3ε expression on T cells via the production of H_2_O_2_ (28, 34). By this way, Th17 cells display an impaired signaling pathway downstream of the T-cell receptor (TCR), leading to inappropriate proliferation and reduced IL-2 production upon TCR triggering (28, 34). In addition, high IL4I1 expression in Th17 cells induces up-regulation of Tob1, a member of the Tob/BTG anti-proliferative protein family, involved in the negative control of the cell cycle (35).

It has also been recently described that Th17 cells show reduced IL-2 responsiveness since they express Musculin (MSC), a member of the basic helix-loop-helix transcription factors, dependent by ROR-γT, which negatively regulates the phosphorylation level of STAT5B upon IL-2 signaling (36). In agreement with these findings, both IL4I1 and MSC were found to be selectively expressed by CD161+ T cells obtained from SF of oligoarticular JIA inflamed joints (34, 36).

It has also been demonstrated that the development of JIA and of other autoimmune diseases depends not only on the amount and the phenotype of Th effector cells, but also on their balance with Treg cells (37). Indeed, several papers demonstrated an accumulation of Th17 and Treg cells in SF and PB of JIA patients (31, 38, 39), in particular in active versus inactive JIA (31), hypothesizing that the joint inflammatory status persists despite the high frequency of Treg cells because Th17 cells show a reduced susceptibility to their regulatory function. This could be also related to the low proliferation rate of Th17 cells (33–36).

## Role of Th17 Plasticity in the Pathogenesis of Oligoarticular JIA

**Figure 1:**
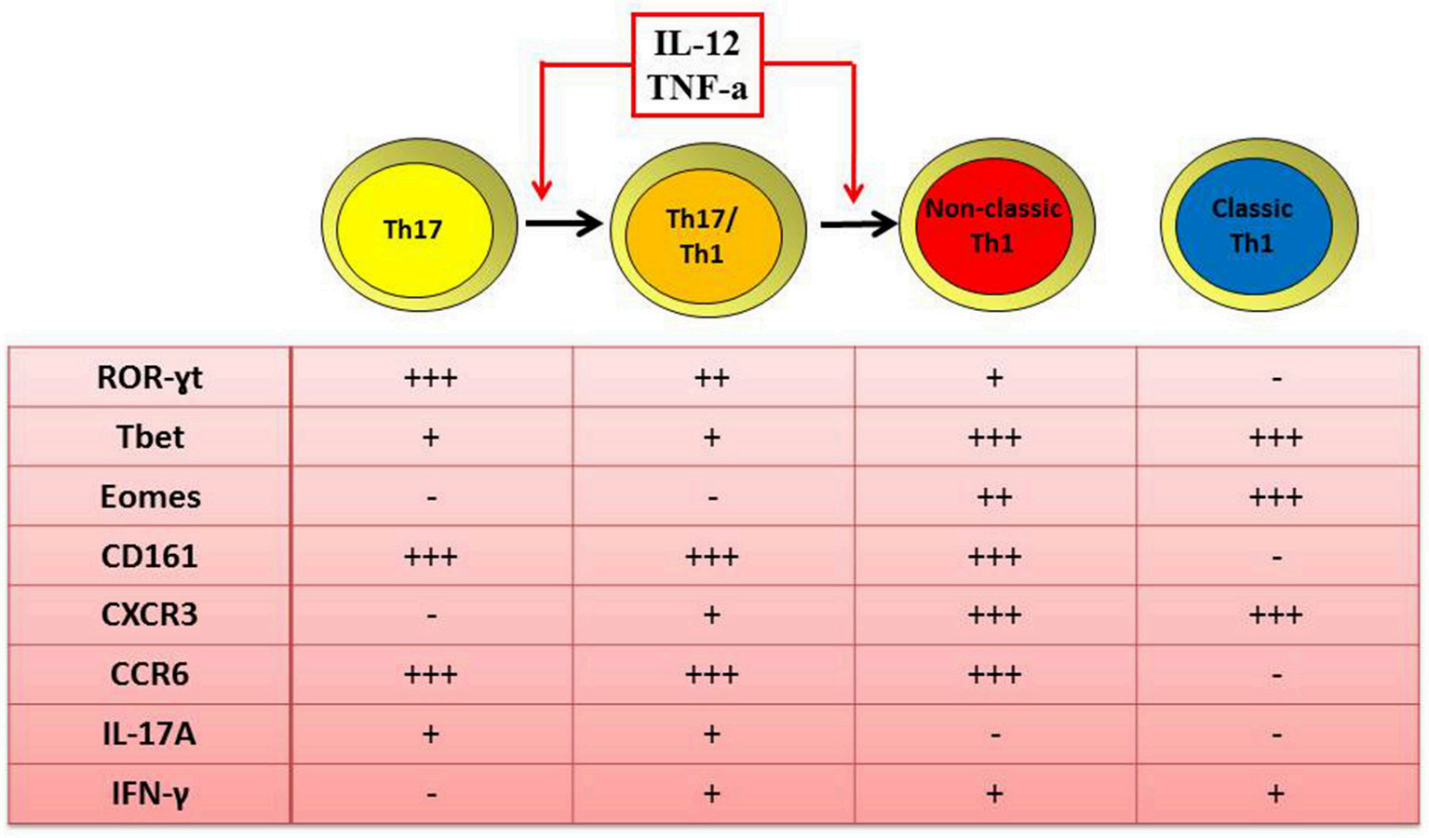
Similar and distinct features of human Th1 and Th17 cells. Both classic and non-classic Th1 cells produce IFN-γ, express the transcription factor T-bet and Eomes and the surface marker CXCR3. Differently from classic, non-classic Th1 cells maintain also the expression of ROR-γT, CD161, and CCR6 as typical transcription factor and surface markers of the Th17 phenotype. In fact, non-classic Th1 cells originate from the shift of Th17 toward the Th17/Th1 phenotype when in the presence of IL-12 and/or TNF-α.

A second explanation for human Th17 cells rarity at inflamed tissues is their phenotype plasticity (28). Indeed, in presence of local inflammatory cytokines such as IL-12 and TNF-α, Th17 cells acquire the ability to produce IFN-γ. At a first stage, cells display an intermediate phenotype known as Th17/Th1 and produce both IFN-γ and IL-17, but they can also rapidly loose IL-17 secretion and become IFN-γ single producers (12, 18, 40). These Th17-derived Th1 cells are defined as non-classic Th1 cells because, differently from classic Th1 cells, they maintain the expression of ROR-γT, CD161, and CCR6 (17, 18, 28, 40), typical molecules of the Th17 subset (Figure 1).

Th17/Th1 and non-classic Th1 cells were found to be enriched in the SF of oligoarticular JIA children compared to their peripheral blood (18, 40). Moreover, a positive correlation between the frequencies of CD4+CD161+ Th17/Th1 cells in the SF of inflamed joints and disease activity parameters was described (18, 28). The shift of Th17 cells toward the non-classic Th1 phenotype is driven by IL-12, that has been found to be elevated in the SF of oligoarticular JIA patients (18, 40). Moreover, the finding that SF-derived Th17 clones share similar TCR Vβ spectra with Th1 CD161+ clones but not with Th1 CD161- ones (18, 40), strongly supported the data of the shift of Th17 toward non-classic Th1 cells. These findings are in agreement with several studies describing an accumulation of Th1 cells in the SF of JIA patients (25, 26), characterized as Th cells producing IFN-γ but without any distinction between the classic and non-classic phenotypes Additional data at epigenetic level confirm that non-classic Th1 cells originate from Th17 (41); indeed it was observed that non-classic Th1 cells exhibit demethylation of RORC2 and IL17A genes, as reported for Th17 cells, whereas classic Th1 cells are completely methylated at these loci (41).

Th17 plasticity consists not only in the acquisition of the ability to produce IFN-γ, but also GM-CSF (42). In fact, it has been described an enrichment of GM-CSF producing T cells with a non-classic Th1 phenotype in the SF of oligoarticular JIA patients (42) and induced *in vitro* by IL-12. This data suggests a possible involvement not only of IFN-γ but also of GM-CSF in JIA pathogenesis, and, accordingly, a positive correlation between GM-CSF protein levels in the SF and the serum parameters of disease activity was described (42).

Moreover it has been recently described that human non-classic Th1 cells development is promoted by the transcription factors Eomes (43), which induces and reinforces IFN-γ production, maintains the Th1 phenotype stability by inhibiting and preventing the re-expression of ROR-γT and IL-17A and promotes GM-CSF secretion (43). Finally, it was shown that Eomes induces, *in vitro*, the production of IFN-γ and GM-CSF by human CD4+ T cells and that cells with this cytokines profile were enriched in the SF of inflamed joints of children with oligoarticular JIA (43). All these data further support the pathogenic role of Th17-derived non-classic Th1 cells.

Additional evidence of the pathogenic features of CD4+CD161+ T cells, including pure Th17 cells and their derivative phenotypes Th17/Th1 and non-classic Th1, derived from a recent paper (44), describing that all these subsets express CHI3L1. This chitinase-like protein without enzymatic activity is defined in the literature as a well-known marker of disease activity and inflammation in several immune-mediated disorders (44–47): its levels are elevated in SF of children affected by oligoarticular JIA and positively correlated to inflammatory parameters (44).

These data of Th17 plasticity, partially solve the literature debate on the pathogenic or protective role of Th17 cells in immunomediated disorders (48, 49), supporting the hypothesis that, at least in JIA, the Th1 subset, both classic and non-classic, is directly involved in the active phase of the disease when the clinical manifestations are evident, and that the pure Th17 cells acquire a pathogenic feature when they start to produce IFN-γ shifting toward the Th17/Th1 and the non-classic Th1 phenotype (18, 20, 40). Anyway, it cannot be excluded that even pure Th17 cells may play a key role in the pathogenesis of JIA during the onset and/or the early-phase of the disease.

## Cross Talk Between T Helper Cells and Resident Synovial Cells

CD4+ T helper lymphocytes orchestrate both RA and JIA chronic inflammation producing cytokines that initiate and maintain the process of synovial proteolysis and proliferation as well as the angiogenesis related to the inflammatory status (50, 51). Synovial fibroblasts (SFbs) are the main tissue resident cell population in the synovia and it has been demonstrated that in adult RA SFbs produce cytokines and matrix-degrading enzymes, crucial to promote cartilage destruction and to mediate inflammation (28, 52). In particular, it has been reported (51) that SFbs derived from SF of oligoarticular JIA patients express high levels of CD106 (VCAM), a sialoglycoprotein which mediates leukocyte-endothelial cell adhesion and signal transduction (51), and whose upregulation is critical to favor leukocytes retention in the inflamed synovia (51). SFbs also showed a peculiar morphology consisting in polygonal cell body, large, and oval-shaped nucleus, many slender protrusions and branches extended out of the cell body (51, 53). This peculiar phenotype of oligoarticular JIA-derived SFbs, was resembled *in vitro* stimulating healthy-derived SFbs with culture supernatants from activated classic and non-classic Th1, but not from Th17, lymphocytes. Indeed, also in these experimental conditions SFbs upregulated CD106 expression and underwent morphological changes (50). It has been demonstrated that TNF-α is the main cytokine involved in this process and that IFN-γ exerts a synergic effect (51, 54). The concept that cytokines produced by T cells play an important role on the activation of SFbs has been confirmed also by the paper of Lavocat et al. (55). It demonstrates with *in vitro* experiments that IL-17A and TNF-α alone are able to induce the expression of IL-6 and IL-8 (55) by both endothelial cells and synoviocytes (even if with different kinetics on each cell type), and that a synergistic effect can be achieved from the use of both cytokines (55). Similar results were obtained also by stimulating endothelial cells and synoviocytes in the presence of culture supernatants from activated T cell clones or recombinant cytokines. Indeed, the main increase in IL-6 and IL-8 production was observed when cells were cultured in presence of supernatants from Th17/Th1 T cell clones that contained both IL-17A and TNF-α (55). The early expression of IL-8 in inflamed joints, directly produced also by Th17 cells itself (9), might explain the massive neutrophil recruitment in the acute phase (56). On the other hand, IL-6 production might be important to sustain the pro-inflammatory process since it is involved in the differentiation and expansion of Th17 cell (57), in VEGF production [thus mediating angiogenesis (58)], as well as in antibody production (59) and in osteoclast activation (55).

The IL-17 signature, which is typical of JIA, is important also for bone and cartilage erosion. In fact, it has been demonstrated that IL-17A acts on SFbs increasing the expression of different types of matrix metalloproteinases, MMP-1, MMP-3 (60). Finally, it is important to note that IL-17A production is not strictly associated to Th17 cells, since it is produced also by additional cells of the immune system enriched in SF of JIA patients, such as CD3+CD8+ and CD3+CD4-CD8- T cells (17, 61, 62) and innate lymphoid cells (62). Collectively, these data suggest that mechanisms actively contributing to joint inflammation in the synovia of JIA patients depend on the final balance and cross-talk between tissue resident cells and immune cells from both the adaptive and innate immune systems.

## Effects of Biological Drugs in the Treatment of JIA: *ex-vivo* and *in-vitro* Observations

Cytokines produced by immune cells (in particular T cells and monocytes) and by tissue resident cells in the synovia contribute to the development of JIA and are responsible for most of the clinical manifestations of the disease. In this view, pro-inflammatory cytokines represent a key therapeutic target for biological treatment. The drugs mainly used and effective in JIA inhibit the activity of TNF-α, IL-1, or IL-6. TNF-α has pleiotropic effects in the inflamed environment of affected joints, acting on different cell populations (51): TNF-α mediates monocyte, macrophage and SFb activation, and it is also responsible for inflammation induction, cartilage degradation, bone erosion and tissue damage (51). Moreover, as previously stated, TNF-α acts on SFbs inducing the upregulation of CD106, thus favoring leukocytes retention within the synovia and increasing joint inflammatory status (51). TNF-α is also involved in the neovascularization process, leading to synovial membrane growth, and in the process of osteoclast-containing 'pannus' formation (51). Additionally, TNF-α interferes with T helper cells phenotype plasticity, mediating the shifting of Th17 lymphocytes toward non-classic Th1 cells (20, 51). Nowadays, JIA patients are treated with non-steroidal antiinflammatory drugs, corticosteroids, and disease modifying antirheumatic drugs including TNF-α antagonists (28). Among these antagonists etanercept is a soluble dimeric fusion protein binding soluble TNF-α. Etanercept has been reported to induce improvement of clinical symptoms (as measured by radiological progression and laboratory parameters of disease activity) in patients affected by immune-mediated arthritis, including RA, JIA, and psoriatic arthritis (28, 63). Moreover, etanercept efficacy in JIA treatment has been demonstrated in randomized clinical trials, as well as in long-term observational registries (28, 64). These clinical effects were sustained by its well-known anti-inflammatory properties on the innate and adaptive immune responses. Moreover, it has been reported a new mechanism of action of etanercept, defining its inhibitory role in the plasticity of Th17 cells toward the non-classic Th1 phenotype mediated by TNF-α (18, 20, 28). In fact, etanercept reduces the proportion of circulating non-classic Th1 cells (20), supposed to play a key role in the pathogenesis of oligoarticular JIA and leads to an increased frequency of Th17 cells (20). Similar evidence was observed also during the treatment of RA patients with adalimumab, a fully humanized monoclonal IgG1 antibody against TNF-α (65), supporting again the important role of this cytokine in the pathogenesis of such diseases. In fact, after 12 weeks of treatment an increase was found in the frequency of IL-17A producing cells that significantly correlated with a reduction in joint inflammation (65, 66). These data support again the important role of this cytokine in the pathogenesis of such diseases. Moreover, it was demonstrated that etanercept acts also on the regulation of CD106 expression on SFbs, in fact *in vitro* administration of this drug negatively interferes with the ability of both classic and non-classic Th1cells supernatants to significantly induce CD106 expression on SFbs (51). Taking into account the important role of CD106 expression on SFbs to mediate leukocytes adhesion, these recent data define also the role of etanercept in interfering with the adhesion of immune cells on SFbs. Even if these data were obtained with *in-vitro* experimental models (51), they may suggest that the reduction of inflammatory cells in the synovia, occurring during etanercept treatment of oligoarticular JIA, may be driven by the reduced retention of immune cells within inflamed joints.

All these data define the immunomodulatory properties of TNF-α inhibitors, especially of etanercept and adalimumab, which could further explain its disease-modifying effect in JIA (20, 51, 66). Regarding infliximab (a human–mouse chimeric anti-TNF-α antibody) and golimumab (a fully humanized monoclonal anti-TNF-α antibody), their use is related mainly to polyarticular JIA and reported in case reports and open-label trials (22).

Among additional drugs inhibiting inflammatory cytokines, the IL-6 receptor antagonist (tocilizumab) and the IL-1 antagonists (anakinra, canakinumab and rilonacept), are currently used in systemic JIA. Anyway these drugs were also tested in clinical practice for oligoarticular or polyarticular JIA (22, 67), and their efficacy may be due, at least in part, to the interference with Th17 expansion and differentiation in the synovia mediated by IL-1 and IL-6.

Moreover, the use of anti-IL-12/IL-23 p40 inhibitors could improve the course of JIA, since both cytokines are involved in Th1 and Th17 differentiation and are important regulators of Th17 plasticity. Ustekinumab, the human monoclonal antibody anti- p40 subunit, is often used in the treatment of psoriatic arthritis and ankylosing spondylarthritis in adults and children (68) and its use in oligoarticular JIA is poor. These data suggest that although these cytokines contribute to joint inflammation, they may not be the principal factors responsible and it is likely that other key mediators are involved (i.e., TNF-α).

Similarly, the use of anti-IL-17A in the treatment of JIA has been explored. In fact data from clinical trials show that secukinumab, the high-affinity fully human monoclonal antibody neutralizing the activity of IL-17A, can be effective in the treatment of JPsA and ERA, suggesting that IL-17 cytokine and Th17 cells play a key role in the pathogenesis of these subtypes of JIA and their role is instead marginal in oligoarticular JIA (18, 20, 40). These data suggest that the use of the appropriate treatment and its effectiveness are related to the different biological conditions found in the different subtypes of JIA (69).

Finally, since oligoarticular JIA is mainly considered to arise due to a dysregulated adaptive immunity, involving Th1 and Th17 effector cells and Treg cells, abatacept was used for its treatment. Abatacept is a chimeric CTLA4 and IgG Fc fusion protein, that, binding to CD80/86 molecules instead of CD28, reduces T helper cells activation. In the treatment of oligoarticolar JIA, it has been demonstrated that abatacept reduced proliferation of CD4+ T cells and their cytokines production (mainly IFN-γ and TNF-α) (21) and reduced the induction of Ig production by B cells (70).

## Conclusion

**Figure 2:**
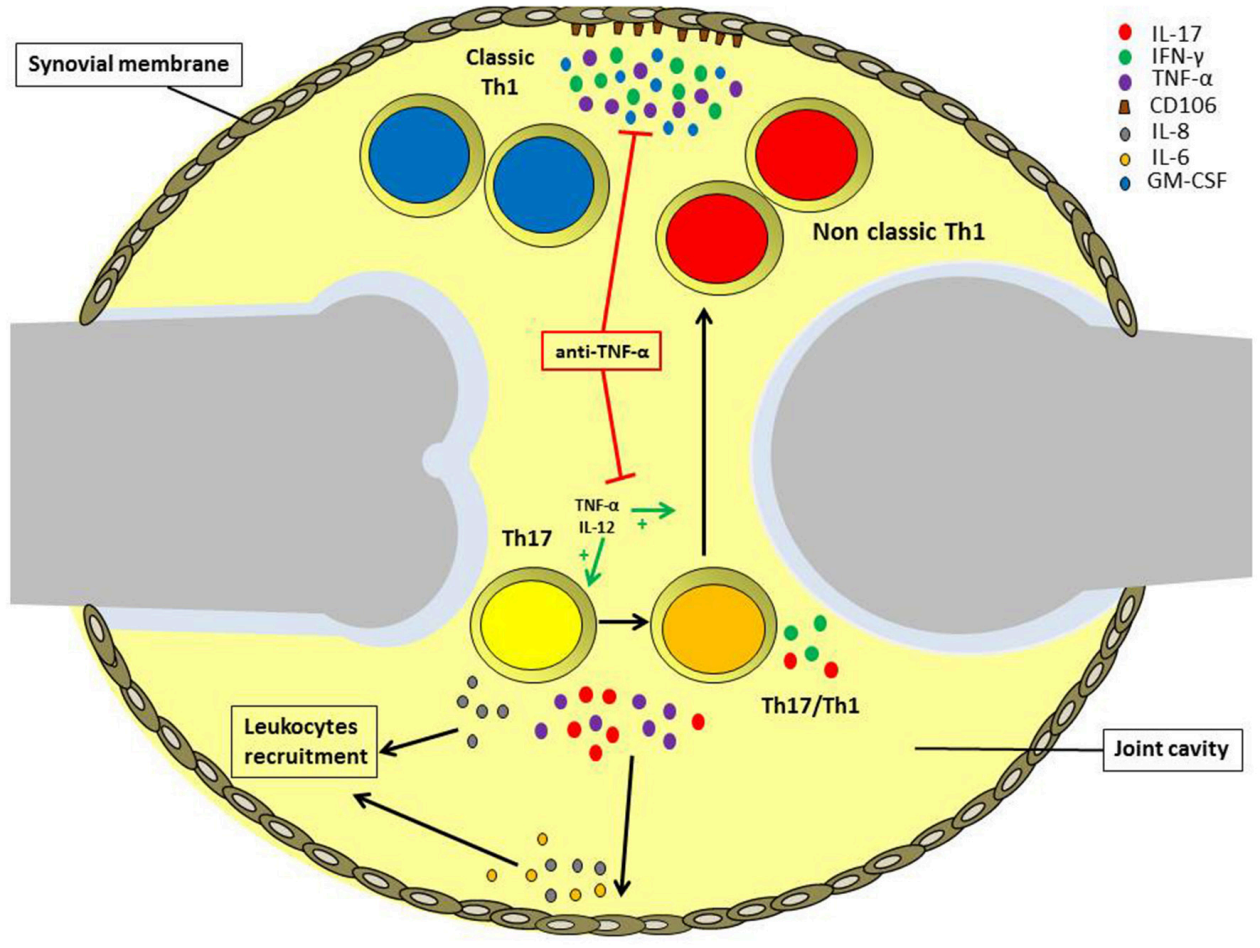
Role of different T helper cell subsets in the pathogenesis of oligoarticular juvenile idiopathic arthritis. IL-17A and TNF-α produced by Th17 and Th17/Th1 cells infiltrating the inflamed joints of oligoarticular juvenile idiopathic arthritis patients, promotes IL-6 and IL-8 release by endothelial cells and synoviocytes in the synovial membrane. These cytokines, in turn, maintain joint inflammation and induce leukocytes recruitment. Inflamed joints are also characterized by high IL-12 and TNF-α levels, which promote Th17 cells phenotypic shift toward Th17/Th1 and non-classic Th1 cells. Non-classic Th1 cells proliferate at higher rate than Th17, and together with classic Th1 cells secrete IFN-γ and TNF-α, favoring the expression of the adhesion molecule CD106 by synoviocytes. Anti-TNF-α treatment can reduce inflammation via both impairing Th17 cell phenotypic shift and also inhibiting CD106 upregulation on the synovial membrane.

In this review, we analyzed the role of different types of T helper cell subsets in the pathogenesis of JIA with particular attention to Th17 and Th1 phenotype (Figure 2). Both Th1 and Th17 cells are critical for the pathogenensis of the disease: Th1 lymphocytes through the production of pro-inflammatory cytokines IFN-γ and TNF-α; Th17 lymphocytes thanks to the shift toward the non-classic Th1 phenotype. Th1 cells subsets exert their function through inducing the expression of CD106 on SFbs, which is crucial in mediating immune cells retention in inflamed synovia. Intriguingly the TNF-α inhibitors are the main biological drugs used in JIA and interfere both with the shift of Th17 to Th1 cells and the TNF-α mediated CD106 upregulation on SFbs. All these data give an explanation at both cellular and molecular level for the efficacy of etanercept treatment in JIA and represent the beginning for further investigation with the aim to identify more specific therapeutic targets. In this view, it is important to underline that different subtypes of JIA are characterized by different inflammatory conditions, whose characterization is crucial for the choice of the efficacious biological treatment.

## Author Contributions

LM and LC wrote the paper. LM and AM prepared the figure. RC, FL, FA, and LC revised the manuscript.

### Conflict of Interest Statement

The authors declare that the research was conducted in the absence of any commercial or financial relationships that could be construed as a potential conflict of interest.
